# Preclinical analysis of human mesenchymal stem cells: tumor tropism and therapeutic efficiency of local HSV-TK suicide gene therapy in glioblastoma

**DOI:** 10.18632/oncotarget.27071

**Published:** 2019-10-22

**Authors:** Lasse Dührsen, Sophie Hartfuß, Daniela Hirsch, Sabine Geiger, Cecile L. Maire, Jan Sedlacik, Christine Guenther, Manfred Westphal, Katrin Lamszus, Felix G. Hermann, Nils Ole Schmidt

**Affiliations:** ^1^ Department of Neurosurgery, University Medical Center Hamburg-Eppendorf, 20246 Hamburg, Germany; ^2^ Apceth Biopharma, 81377 Munich, Germany; ^3^ Department of Neuroradiology, University Medical Center Hamburg-Eppendorf, 20246 Hamburg, Germany

**Keywords:** glioblastoma, brain tumor, stem cells, gene therapy, migration

## Abstract

Glioblastoma are highly invasive and associated with limited therapeutic options and a grim prognosis. Using stem cells to extend current therapeutic strategies by targeted drug delivery to infiltrated tumors cells is highly attractive. This study analyzes the tumor homing and therapeutic abilities of clinical grade human mesenchymal stem cells (MSCs) in an orthotopic glioblastoma mouse model. Our time course analysis demonstrated that MSCs display a rapid targeted migration to intracerebral U87 glioma xenografts growing in the contralateral hemisphere within the first 48h hours after application as assessed by histology and 7T magnetic resonance imaging. MSCs accumulated predominantly peritumorally but also infiltrated the main tumor mass and targeted distant tumor satellites while no MSCs were found in other regions of the brain. Intratumoral application of MSCs expressing herpes simplex virus thymidine kinase followed by systemic prodrug application of ganciclovir led to a significant tumor growth inhibition of 86% versus the control groups (p<0.05), which translated in a significant prolonged survival time (p<0.05). This study demonstrates that human MSCs generated according to apceth’s GMP process from healthy donors are able to target and provide a significant growth inhibition in a glioblastoma model supporting a potential clinical translation.

## INTRODUCTION

Glioblastoma represent one of the most aggressive brain tumor types with a devastating prognosis of 12–15 months after diagnosis despite intensive treatment including surgery, radiation and chemotherapy [1]. Its highly invasive nature within the brain parenchyma limits surgical resection and recurrence due to remaining infiltrating tumors cells is inevitable [2]. The blood-brain-barrier limits available systemic pharmacological options and glioblastoma molecular diversity and instability often results in the rapid development of therapeutic resistance [3]. The inherent capability of stem and progenitors cells for extensive targeted migration towards brain tumors has the potential to improve drug delivery and to overcome these therapeutic limitations [4].

Preclinical data has demonstrated the efficiency of stem cell based-targeted delivery of various therapeutic payloads to glioblastoma xenografts [5–8]. Clinical proof-of-concept was confirmed in a recently completed safety/feasibility study (NCT01172964) in which a neural stem cell mediated enzyme-prodrug treatment strategy was tested in patients with recurrent glioblastoma [9]. However, one of the major challenges for clinical translation of stem cell-based concepts in neurological disease remain the choice of type and origin of the stem- or progenitor cells [10]. Neural stem- or progenitor cells of the central nervous system pose logistical and ethical problems especially since they are often of embryonal or fetal origin. Other more easily accessible tissue sources such as the bone marrow would be an ideal source for stem cells. Bone marrow-derived mesenchymal stem cells have been demonstrated to be effective in various preclinical cancer models including glioblastoma suggesting promise for future clinical translation [11].

A recently finished phase I, first in human and first in class trial using genetically modified autologous mesenchymal stem cells expressing herpes simplex virus thymidine kinase (HSV-TK) in combination with ganciclovir (GCV) demonstrated acceptable safety and tolerability in advanced gastrointestinal cancer patients [12]. Therefore, we assessed the migratory and tumor homing capabilities of these genetically modified human mesenchymal stem cells in an intracerebral glioblastoma model and evaluated the therapeutic efficiency of local HSV-TK suicide gene therapy.

## RESULTS

### Characterization of human mesenchymal stem cells and transgene expression

Human MSCs were isolated from bone marrow of healthy donors in accordance with apceth’s GMP process [12]. The isolated MSCs were retrovirally transduced with therapeutic vector that constitutively expresses HSV-TK under control of the EFS promoter for *in vitro* and *in vivo* efficacy. Furthermore, cells were transduced with a GFP encoding vector to allow for *in vivo* and *ex vivo* tracking of GFP expressing cells. Subsequently, the transduced cells were purified using puromycin selection, expanded and cryo-preserved.

**Figure 1:**
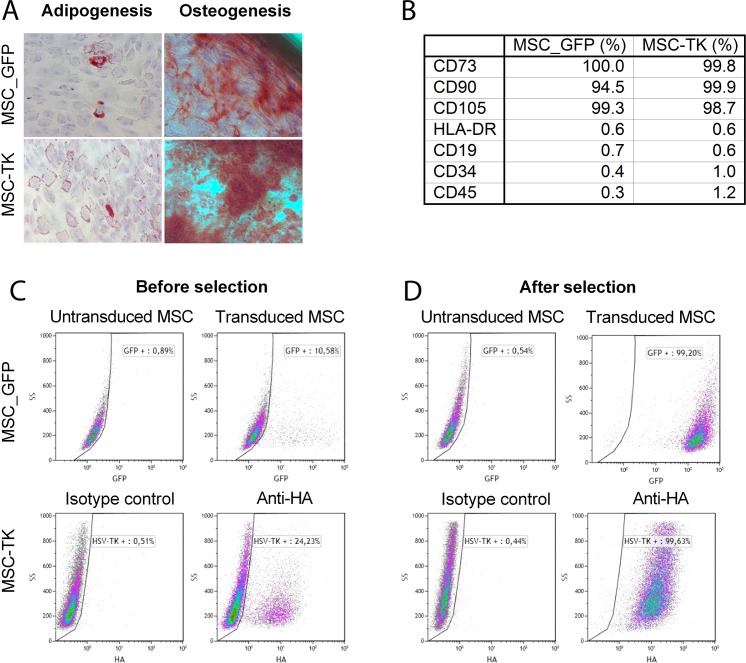
Characterization of transduced MSCs by *in vitro* differentiation assay and flow cytometry. The capacity of genetically modified MSCs to differentiate to adipocytes and osteocytes was confirmed by differentiation assays **(A)**. Percentage of positive surface marker. MSC_GFP and MSC-TK were positive for the MSC markers CD73, CD90 and CD105 and negative for the impurity markers tested (CD19, CD34 and CD45) **(B)**. After transduction with retroviral vectors to express GFP or HSV-TK, 10.6 and 24.2% of cells were transduced before and 99.2 and 99.6% of cells after puromycin selection, respectively **(C** and **D)**. Abbreviation: HA, hemagglutinin.

To ensure an MSC-like identity, the cells were characterized in regards to differentiation capacity, the expression of surface markers and transgene expression. The genetically modified MSCs differentiated into adipocytes and osteocytes (Figure 1A). Both, GFP and HSV-TK expressing MSCs were positive (< 94%) for MSC markers CD73 (100.0%, 99.8%), CD90 (94.5%, 99.9%) and CD105 (99.3%, 98.7%) and negative (< 2%) for impurity markers CD19 (0.7%, 0.6%), CD34 (0.4%, 1.0%) and CD45 (0.3%, 1.2%) as well as HLA-DR (0.6%, 0.6%) (Figure 1B). Flow cytometric analysis revealed 10.6% GFP positive MSC after transduction and 99.2% positive cells after selection. For HSV-TK expressing MSCs, 24.2% of cells were positive before and 99.6% after puromycin selection as determined using an antibody directed to the hemagglutinin-tag (HA-tag) linked to the HSV-TK transgene (Figure 1C, 1D). After thawing 96.15% (MSC-GFP) and 97.69% (MSC-TK) of cells were vital, respectively as determined by Annexin V/7AAD flow cytometry.

### *In vitro* bystander killing depends on gap junctions

Cell that are transduced with HSV-TK are efficiently killed by GCV. The bystander-killing refers to the fact that nearby non-transduced cells are also sensitive towards GCV treatment. It has previously been shown that gap junctions are necessary to allow efficient distribution of phosphorylated GCV between cells, which is a prerequisite for the bystander effect [13, 14].

**Figure 2:**
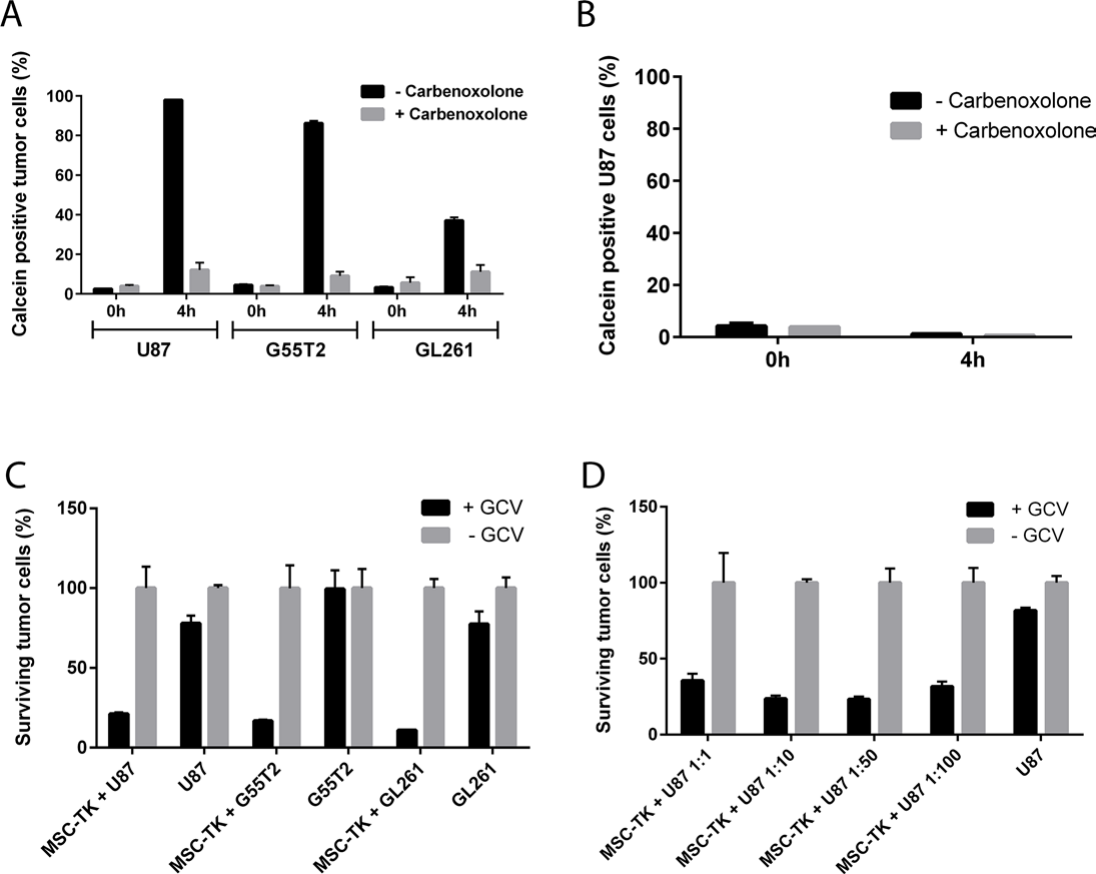
*In vitro* gap junction formation and bystander killing of glioblastoma cells by HSV-TK expressing MSCs. Dye transfer of Calcein stained MSCs to glioblastoma cells indicate efficient gap junction formation **(A** and **B)**. Anti-tumoral efficacy was demonstrated by significant reduction of surviving U87, G55T2 and GL261 tumor cells after MSC-HSV-TK coculture and GCV co-treatment **(C)** even with at low M:T ratios up to 1:100 **(D)**.

A dye transfer assay was performed to demonstrate gap junction formation between MSC_HSV-TK and different glioblastoma cell lines (U87, G55T2 and GL261). Efficient transfer of gap junction permeable dye Calcein AM to CMTPX (cell tracker red) negative tumor cells 4h after coculture (U87 97.9+/-0.0%, G55T2 86.2+/-1.2%, GL261 37.0+/-1.7% Calcein positive tumor cells) was observed which could be inhibited by gap junction inhibitor Carbenoxolone (Figure 2A). It was further confirmed, that the dye transfer is cell-cell contact dependent, since no dye transfer was observed when cells were separated by transwells (Figure 2B).

To demonstrate that genetically modified MSCs that constitutively express HSV-TK are able to kill glioblastoma cells in the presence of GCV, *in vitro* bystander killing assays were performed. MSC_HSV-TK were cocultured with CMFDA (cell tracker green) or GFP-labeled U87, G55T2 or GL261 tumor cells at a ratio of 1:1. The cocultures were treated with GCV for three consecutive days before quantitative analysis by flow cytometry to determine the percentage of surviving tumor cells. According to the FACS data obtained, a significant reduction of surviving tumor cells was observed after coculture of HSV-TK expressing MSCs with CMFDA stained U87, G55T2 or GL261 glioblastoma cells and addition of GCV (21.2+/-1.0%, 16.8+/-0.8% or 11.0+/-0.1% surviving tumor cells, respectively) compared to control samples without GCV treatment (Figure 2C). A reduced percentage of vital tumor cells was also observed for U87 and GL261 control samples (without MSC coculture) and the addition of GCV, indicating slightly toxic effects of GCV treatments on these tumor cells (77.9+/-4.9% or 77.5+/-8.0% surviving tumor cells, respectively) (Figure 2C). Lower M:T ratios, up to 1:100, were observed to induce efficient killing of U87 target cells after addition of GCV prodrug (1:10 23.7+/-2.0%, 1:50 23.3+/-1.8%, 1:100 31.7+/-3.3% surviving U87 cells). Hence, the *in vitro* data indicate an efficient anti-tumoral effect of MSC_HSV-TK against glioblastoma cells, even at low M:T ratios (Figure 2D).

### MSC display tumor targeted migration *in vitro*

**Figure 3:**
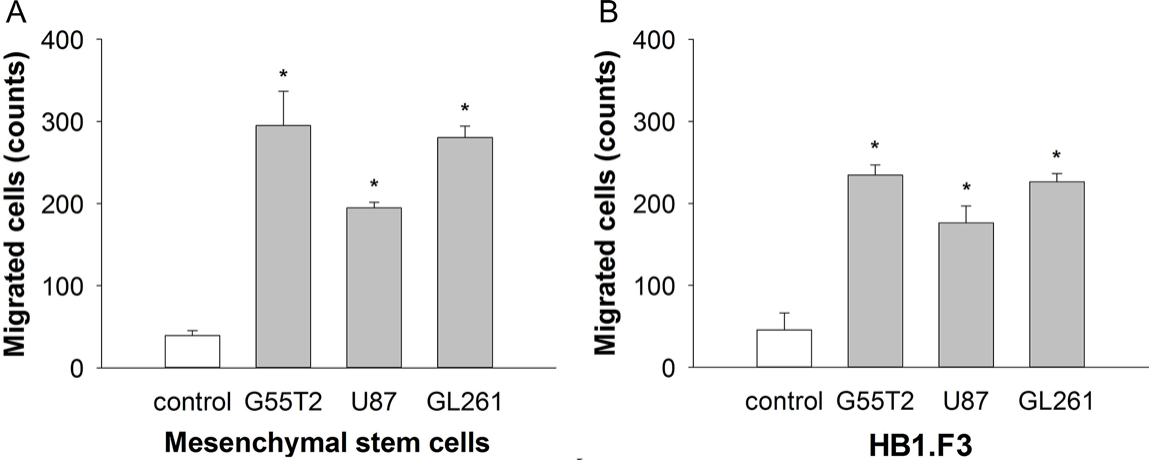
Mesenchymal stem cell (MSC) migration **(A)** in response to conditioned media from glioblastoma cell lines in a 96-well modified Boyden chamber assay in comparison to the well-established human neural stem cell line HB1.F3 **(B)**. Conditioned media from all tumor cell lines significantly stimulated the directional migration of the MSCs compared to the control (^*^ *p* < 0.001, *t* test).

MSCs are useful vehicles for the delivery of therapeutic genes to tumors because of their ability to specifically migrate towards tumor tissue [11]. To confirm this tumor tropism *in vitro*, a modified Boyden chamber assay was performed. Conditioned medium collected from different glioblastoma cells (G55T2, U87 and Gl261) was used as an attractant of MSC. Compared to unconditioned medium (40+/-6 migrating cells), conditioned medium collected from G55T2, Gl261 and U87 led to a 7.4-fold (295+/-42 migrating cells, p<0.01), 7-fold (281+/-14 migrating cells, p<0.01) and 4.9-fold (195+/-7 migrating cells, p<0.01) increase in migration, respectively (Figure 3A). In addition, the human neural stem cell line HB1. F3 was assessed in the modified Boyden chamber assay. Compared to the basal migration rate of 46+/-21 cells induced by unconditioned medium, medium collected from G55T2, Gl261 and U87 glioblastoma increased migration 5-fold (226+/-20, p<0.01), 4.7-fold (216+/-22, p<0.01) and 3.7-fold (171+/-20, p<0.01), respectively (Figure 3B). HB1.F3 has been previously described as a cell line that very efficiently homes to glioblastoma xenografts [15, 16]. Compared to the results achieved with HB1.F3, the human MSC demonstrated a similar pattern for a targeted migration towards glioblastoma.

### Human MSC efficiently target glioblastoma xenografts after intracranial injection

**Figure 4:**
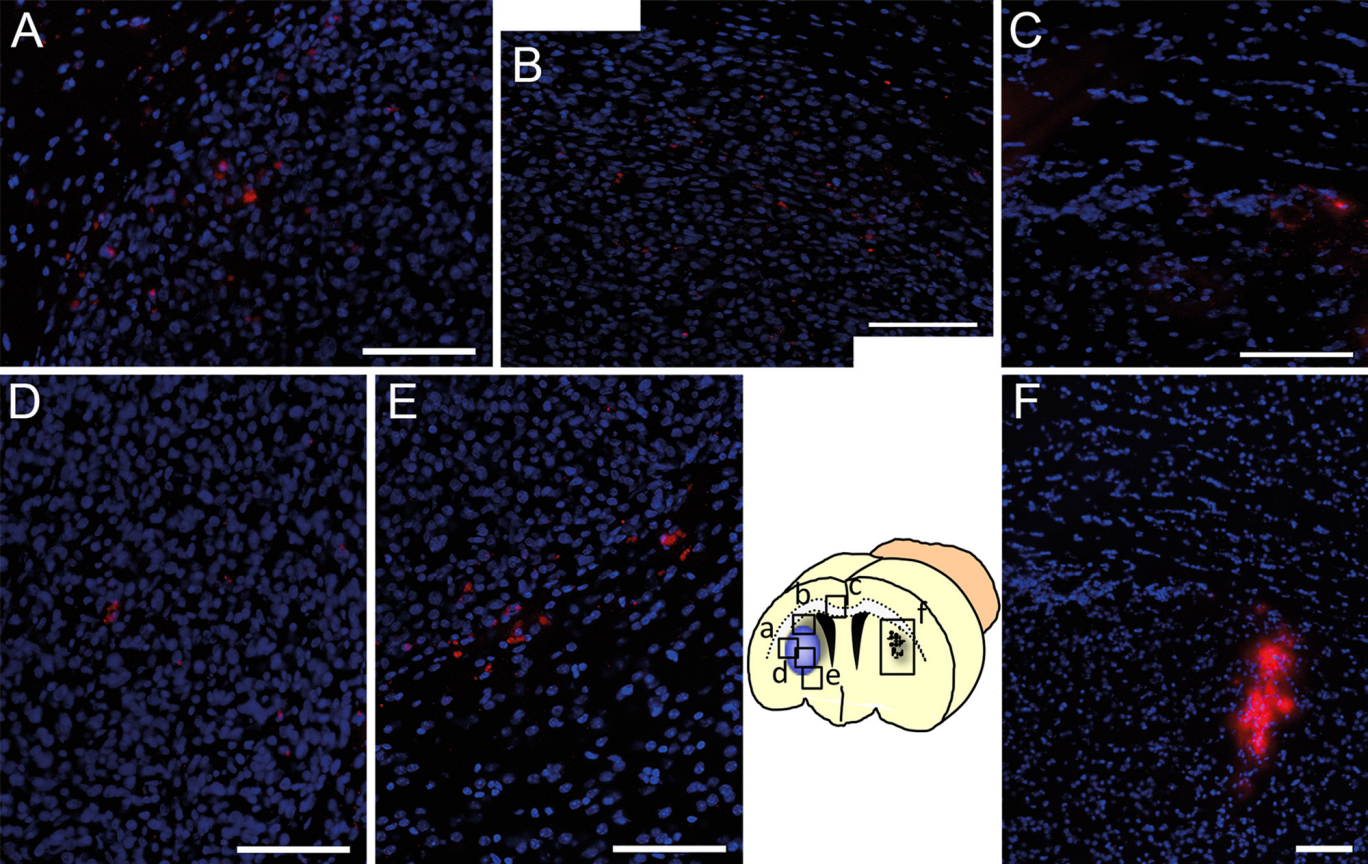
Tumor targeted *in vivo* migration of DiI-labeled MSCs (red) seven days after stereotactic injection into the contralateral hemisphere **(F**, injection site) of a growing U87 glioblastoma xenograft. Within seven days DiI-MSCs were found in the tumor area **(A** and **B)** after migration towards the glioblastoma in the contralateral hemisphere via the corpus callosum **(C)**. Even in distant parts of the tumor DiI-labeled MSCs were found intratumorally **(D)** or peritumorally **(E)** whereas no MSCs were found in other regions of the brain. Scale Bars: 100 μm.

The next aim was to confirm the tumor tropism of human MSCs in an *in vivo* model. Human U87 glioblastoma cells were injected into the right forebrain of mice to establish tumors. Ten days later, 5×10^4^ DiI-labeled MSCs from cell culture were injected into the left forebrain using the matching contralateral coordinates as used for the tumor induction (n=5 mice). Seven days later, histological analysis demonstrated a directed migration of MSCs from the injection site via the corpus callosum towards the tumor in the contralateral hemisphere. DiI-positive MSCs were found preferably at the tumor-parenchyma border encircling the tumor but also invading into the tumor mass. Serial sections of the whole brain did not reveal any DiI-positive MSC in other distant cerebral or cerebellar regions confirming a targeted tumor tropism (Figure 4).

**Figure 5:**
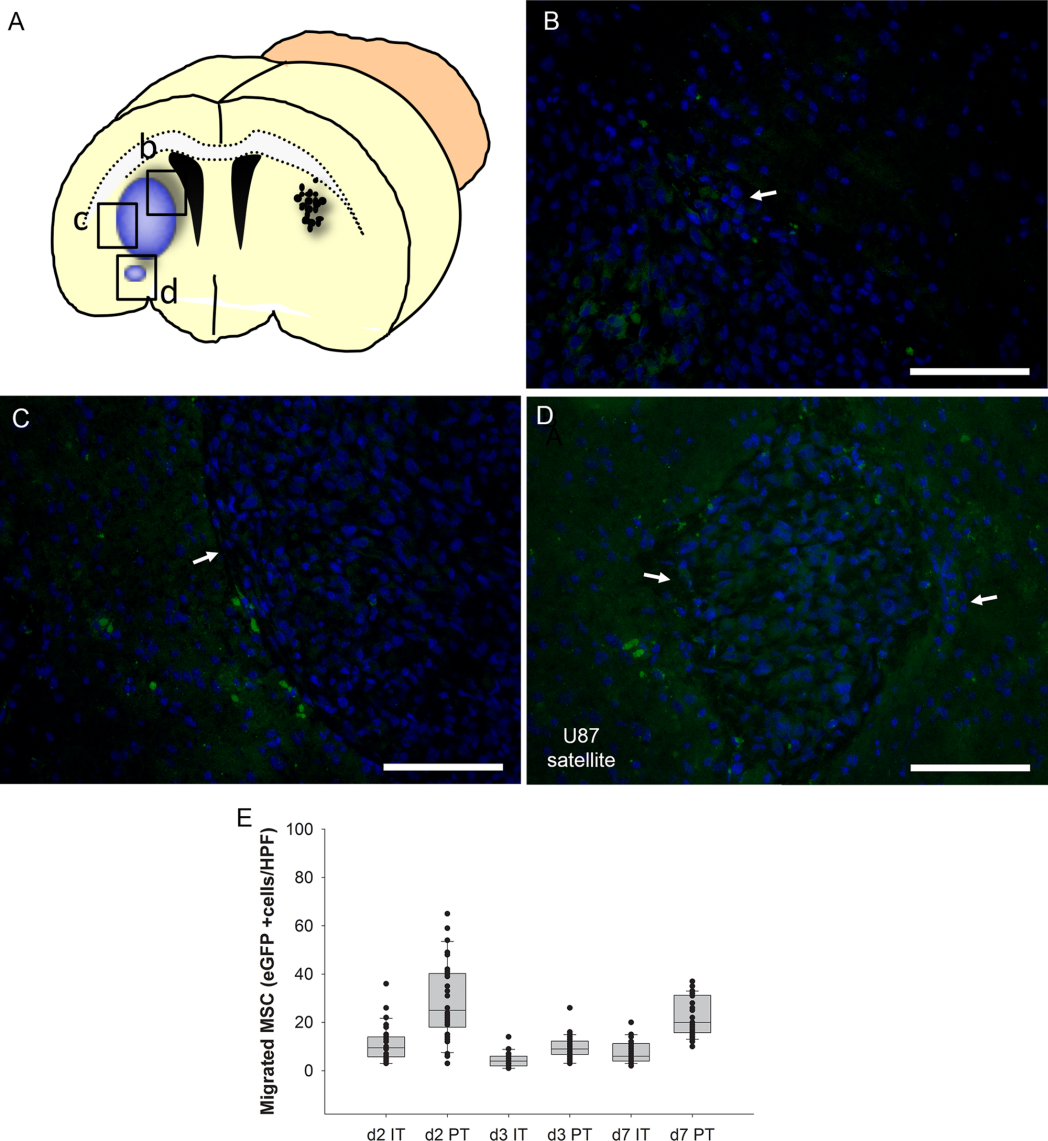
Time course analysis of intratumoral (IT) and peritumoral (PT) accumulation in the intracerebral human U87 glioblastoma model after injection of eGFP-expressing MSCs (green) into the contralateral hemisphere **(A)**. Two days after injection MSCs enriched at the tumor site with intratumoral **(B)** and peritumoral **(C)** hot spots. Even a tumor satellite distant from the main tumor mass was peritumorally encircled by MSCs **(D)**. The majority of MSCs was found in the immediate peritumoral area after two days **(E**, box whisker chart**)**. Arrows = tumor border. Scale Bars: 100 μm.

In order to assess the spatial and temporal dynamics of MSC migration, we performed a histological time course analysis. Ten days after U87 human glioblastoma cell injection into the right forebrain, 15 mice received a stereotactic injection of 2x10^5^ freshly thawed eGFP-expressing MSCs to the contralateral hemisphere. Groups of mice (n=5 each) were sacrificed 2, 3 and 7 days after MSC application and serial sections of the whole brain were histologically evaluated to detect MSCs (Figure 5A). Within the first two days MSCs were able to reach the tumor in the contralateral hemisphere, migrated into the tumor mass (Figure 5B) and accumulated in the peritumoral area directly at the brain parenchyma-tumor border (Figure 5C). Even a small tumor satellite distant from the main tumor mass was targeted by the MSCs (Figure 5D). Quantification of migrated GFP-expressing MSCs revealed that the tumor homing occurred within the first two days with the majority of cells localized peritumorally (PT) when compared to intratumorally (IT) localized GFP-positive cells (PT 25+-2.9 cells/hpf vs IT 9.5+-1.3 cells/hpf, p<0.001 MWU-test)(Figure 5E). The number of IT- or PT-localized MSCs was not significantly different between day 2 and day 7 (d2 IT 9.5+-1.3 cells/hpf vs d7 IT 6+-0.9, p=0.074; d2 PT 25+-2.9 cells/hpf vs d7 PT 20+-1.4, p=0.12). The results demonstrate that MSCs are able to migrate in a targeted manner to glioblastoma and small tumor satellites distant from the main tumor mass making them ideal delivery vehicles for therapeutic genes.

### MSC migration can be tracked *in vivo* by non-invasive MR imaging

**Figure 6:**
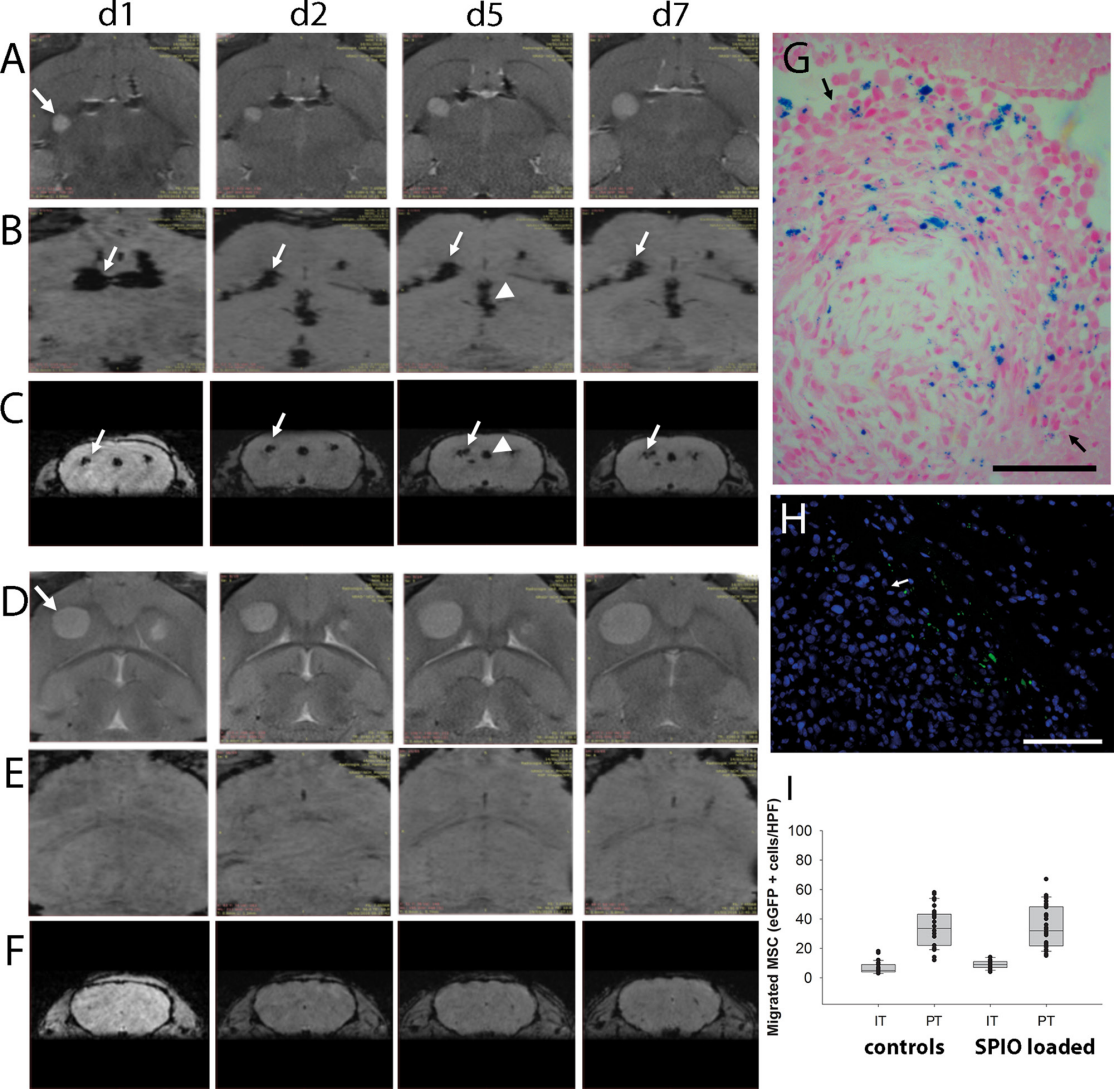
*In vivo* MR-imaging of tumor targeted migration of human MSCs towards intracerebral U87 glioblastoma xenografts (A–C = SPIO-loaded MSCs, D–F = control animals with non-loaded MSCs). Axial sections of T2-weighted MR images demonstrate glioblastoma growth (arrow) from day one (d1) to day seven (d7) after MSC injection into the contralateral hemisphere (**A**, d1-d7; D, d1-d7). Signal voids in axial **(B)** and in coronal **(C)** sections of SW images indicated SPIO-labeled MSCs migrating from the injection site (arrows) via the corpus callosum towards the tumor site over time. Signal voids in the ventricular system (arrowhead, e.g. D/d5 and C/d5) indicated MSC presence and **(G)** histological analysis confirmed tumor spread into the ventricular system where presence of SPIO-loaded MSCs within the tumor mass (arrows) was confirmed by Prussian blue staining (blue). **(E, F)** Corresponding SW images of control mice which received MSC without SPIO loading did not display similar signal voids. **(H)** Tumor tropism of human SPIO-loaded MSCs was furthermore confirmed by the presence of GFP-positive MSCs at the contralateral tumor site (arrow = tumor border). **(I)** Histological quantification of SPIO-loaded and non-loaded GFP-positive MSCs within the tumor and the peritumoral area. Scale Bars: 100 μm.

With regard to future clinical development of MSC-based products, clinical applicable non-invasive methods for monitoring distribution of cellular therapeutics in the patients are highly desirable. Therefore, we tested if MR imaging is a suitable method to allow *in vivo* tracking of MSCs, which have been loaded with a superparamagnetic iron oxide (SPIO) based MRI tracer. Tumors were established as described above. The GFP-expressing L87 MSC cell line loaded with SPIO or without SPIO as controls were injected into the left forebrain at the same location contralateral to the growing tumor (n=6). MR imaging at day 1, 2, 5 and 7 demonstrated that the MSCs reached the tumor area in the contralateral hemisphere already within the first 24 hours (Figure 6). Hypointensive signals in axial SWI across the corpus callosum (Figure 6B) at day 1 indicated a directed migration towards the tumor. In coronal sections (Figure 6C) the hypointensive signals within the tumor area further increased from day 1 to day 7 indicating that further SPIO-loaded MSC reached the tumor. In the control animals with MSCs but without SPIOs, no similar signal hypointensities were observed (Figure 6D, 6E, 6F). MSCs enriched in the tumor which had spread into the ventricular system as indicated by the signal hypointensities in the midline (Figure 6B, 6C, arrowhead). This was confirmed by Prussian blue staining demonstrating blue colored SPIO-loaded MSC within the tumor (Figure 6G). Furthermore, histological sections after day 7 confirmed the presence of GFP-labeled MSCs within the tumor area (Figure 6H). Histological quantification of GFP-positive MSCs within the tumor and the peritumoral area confirmed a similar migration pattern and no significant impact of SPIO loading on the migratory capabilities (Figure 6I). The results indicate that MR imaging is a potential clinically applicable method to monitor biodistribution of MSC-based therapeutics.

### Therapeutic efficiency of HSV-TK expressing MSC on glioblastoma growth and survival

**Figure 7:**
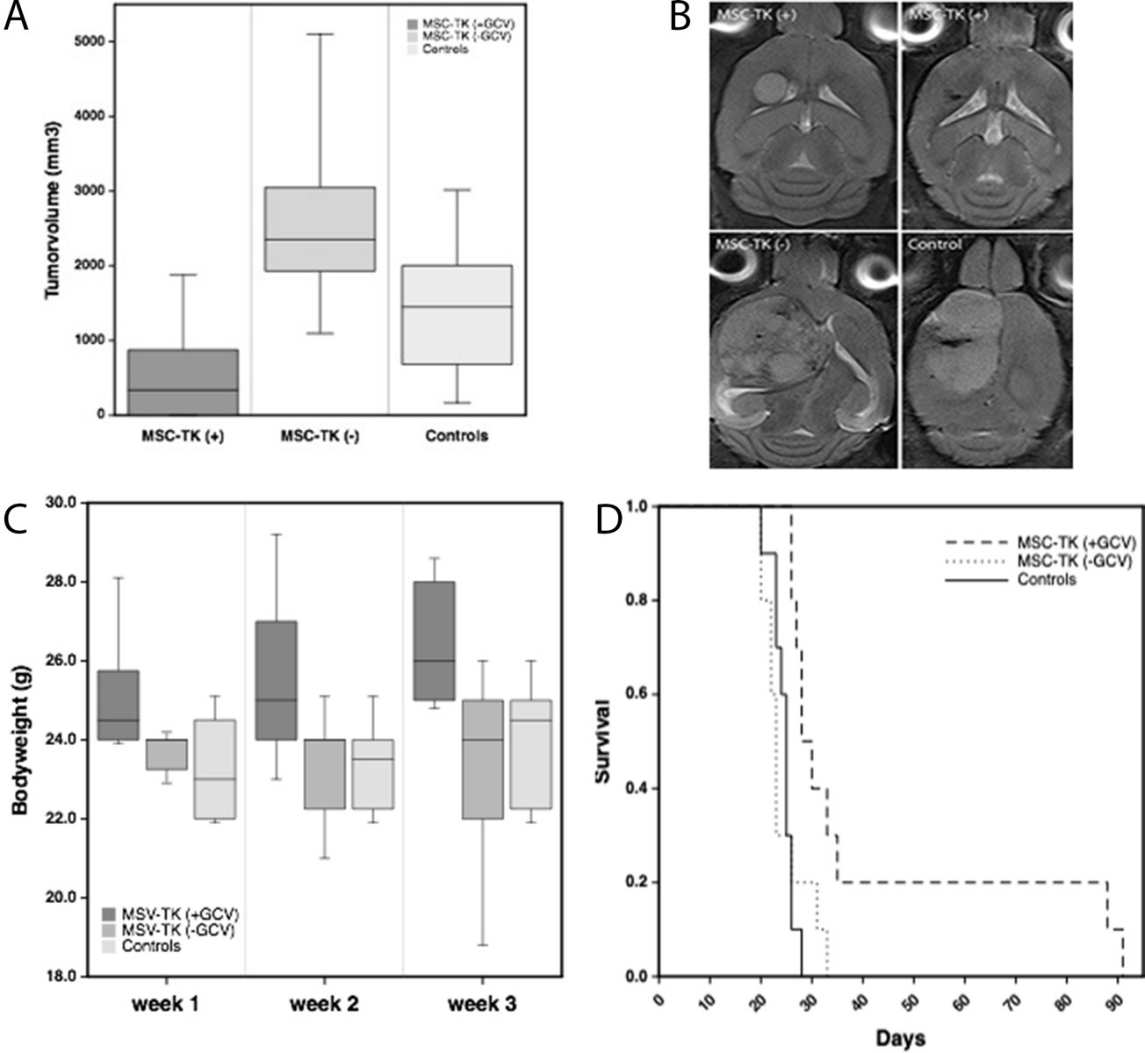
Therapeutic effects of local intratumoral injection of HSV-TK expressing MSC (MSC-TK) followed by five days of systemic GCV administration. **(A)** *In vivo* MR-imaging at day 20 after tumor induction demonstrated significant smaller tumor volumes compared to the control groups (p<0.05). There was no significant difference between the control groups. **(B)** Representative axial T2-weighted MR images of the different treatment groups. **(C)** The body weight of animals treated with MSC-TK followed by systemic application of the prodrug GCV were significantly higher than compared to the controls (Box whisker charts, p<0.05). **(D)** Kaplan–Meier analysis of survival with significantly longer survival of treated animals compared to controls (p <0.05).

After tumor specific homing of MSCs *in vivo* had been established, our aim was to assess the therapeutic efficiency of HSV-TK expressing MSC in an *in vivo* glioblastoma model. For this U87 glioblastoma xenografts were established by stereotactic injection into the right forebrain of 6-week old nude mice as described above. Local intratumoral injection of 0.4x10^6^ MSC_TK followed by five days of systemic GCV administration led to a significant tumor growth inhibition of 86% and 77% compared to the control groups (MSC-TK(-) and NaCl(+)), respectively as assessed by MR imaging at day 20 after tumor cell administration (Figure 7A). Animals who received HSV-TK expressing MSCs followed by GCV administration displayed only small tumors and in five animals hardly any tumor mass was visible (Figure 7B). 3D-quantification based on T2 MR imaging confirmed significant smaller tumor volumes (339.5+/-275 mm^3^) than in the controls that received no GCV after MSC-TK injection (2353+/-950 mm^3^) or that received no cells but GCV (1449+/-331 mm^3^). This therapeutic effect was paralleled by significant higher bodyweights than in the controls that presented rapid tumor growth (Figure 7C). Tumor growth inhibition translated into a significant extend of survival (p<0.05) with a median survival of 29 days compared to the control groups that received MSC-TK followed by GCV administration and those without cells but GCV (23 days and 25 days, respectively) (Figure 7D).

## DISCUSSION

Malignant gliomas are highly invasive and associated with a grim prognosis. Using stem cells to extend current therapeutic strategies by targeted and localized drug delivery to infiltrated tumors cells is highly attractive and furthermore allows the use of additional therapeutic payloads otherwise not applicable by conventional delivery strategies [17]. This study analyzes the tumor homing abilities of human MSCs, produced according to apceth’s GMP process, in an orthotopic glioblastoma mouse model in preparation for potential clinical translation of this concept.

Our data demonstrated a rapid and targeted migration of intracerebrally injected MSCs towards glioblastoma xenografts growing in the contralateral hemisphere as demonstrated by histological and MR imaging-based analyses. Already within 48 hours, MSCs were found to accumulate into the tumor area even in the most distant parts of the tumor and in small distant tumor satellites, whereas no MSCs were found in other regions of the brain. This is in line with a previous study demonstrating that MSCs displayed a targeted migration towards tumors [8]. Migration of stem- or progenitor cells towards single or multifocal areas of neoplastic, ischemic, degenerative or traumatic lesions within the brain have been observed in a variety of animal models [4, 18]. A number of cytokines and its corresponding receptors such as vascular endothelial growth factor (VEGF) [15, 19], stem cell factor/c-kit [20], stromal cell-derived factor-1/CXCR4 [8] or platelet derived growth factor-BB [8] have been identified as chemotactic factors for neural stem cells (NSCs) and MSCs. The extensive upregulation of VEGF in malignant gliomas was able to induce a tumor-targeted and long-range migration of NSCs after intracerebral injection [15, 21]. VEGF demonstrated also significant pro-migratory and pro-invasive effects on human MSCs in a glioma spheroid model [19] but failed to show a similar effect in a Matrigel invasion assay [8]. However, as VEGF is the known major player of angiogenesis in health and disease it also stimulates cerebral endothelial cells to express a multitude of other chemotactic factors potentially guiding stem- and /progenitor cells towards a neuro-pathology [21]. MSCs are able cross the blood-brain-barrier and to enter the brain after direct venous or arterial intravascular injection [8, 22]. Even after noninvasive intranasal delivery MSCs are able to enter the brain via the olfactory mucosa/cribriform plate and migrate towards gliomas [23] as it has been shown with NSCs [7]. The recently discovered meningeal lymphatic system adds to the complexity of potential pathways in and out of the brain and future studies need to unravel its relevance for cell-based therapies in neurological disorders [24].

Our time course analysis demonstrated that the majority of MSCs were localized peritumorally, surrounding the tumors and that the peri- and intratumoral enrichment occurred within the first two days after intracerebral MSC injection. We observed a stable number of MSCs within the tumor region up to day 7, but no further increase of MSC density, which is in agreement with Huang et al. using rodent MSCs in a brain stem tumor model [25]. The *in vitro* analysis comparing the tumor targeted migration of MSCs with the neural stem cell line HB1.F3, which was recently tested in a clinical phase I study in recurrent glioblastoma, revealed a similar tumor tropism towards different glioblastoma cell lines [9, 16]. These findings are in line with a previous comparison of MSCs with the HB1.F3 cell line in a brain stem tumor model [26]. MSCs are multipotent, fibroblast-like cells which can be generated from different tissues including fat, cord blood and bone marrow and easily expanded in culture [27–29]. Their low immunogenic characteristics allow the generation of cell products for allogeneic application [30]. Taken together, these results support the potential clinical use of MSCs as an ethical and logistic ideal alternative to neural stem cells as drug delivery vehicles for the treatment of glioblastoma.

As every novel therapeutic option needs to get integrated in the current standard therapeutic regimen it seems optimal to initiate a stem cell-based therapy in glioblastoma patients directly after resection of the tumor mass. With the number of tumor cells at its minimum, the remaining infiltrated glioma cells can then be targeted by MSCs delivering a therapeutic payload. In this study, we used the well-known HSV-TK suicide gene therapy as a therapeutic paradigm for the MSC-based therapy. The single intratumoral injection of 0.4×10^6^ HSV-TK expressing human bone marrow-derived MSCs followed by five days of systemic GCV administration led to a significant tumor growth inhibition of 86% and prolonged survival in the orthotopic U87 glioblastoma mouse model. This is in line with a previous study using HSV-TK expressing MSCs derived from adipose tissue, which demonstrated a significant tumor growth inhibition in the U87 glioblastoma model. However, the treatment effects were only assessed early after MSC treatment and the effects on survival were not investigated [29]. Our vitro data using different glioblastoma cells lines demonstrated that gap junction intercellular communication of the HSV-TK expressing MSCs contributed to the significant bystander killing of tumor cells as demonstrated previously by Matuskova et al. [14].

The HSV-TK suicide gene therapy was already clinically tested in a randomized, open-label, phase 3 trial in patients with glioblastoma [31]. However, in this trial the suicide gene therapy was mediated by an adenovirus which is not motile and therefore clearly limiting the therapeutic distribution. Although some therapeutic effects were observed, the clinical trial failed as the primary endpoint was not reached [31]. It seems reasonable to speculate that the motile abilities of HSV-TK expressing MSCs have the potential to optimize the therapeutic distribution and may therefore improve therapeutic efficiency. Nevertheless, prodrug-based suicide gene therapies may not be sufficient enough to provide a sustained tumor control in the brain as the therapeutic efficiency also depends on the distribution and concentration of the systemically applied prodrug. Other gene products with a direct therapeutic effect such as TRAIL [32] or interleukin-12 [33] or the simultaneous expression of different therapeutic gene products [34] have been preclinically tested using stem cell-based approaches and may be more effective while also expanding the so far highly limited therapeutic options in malignant glioma.

Our study establishes that our genetically modified human MSCs generated according to apceth’s GMP process from healthy donors are able to target and provide a significant growth inhibition and increase survival in a glioblastoma model. Our data support a potential clinical translation as motile MSCs represent an ideal platform technology for the targeted delivery of various therapeutic gene products in malignant glioma.

## MATERIALS AND METHODS

### Generation and characterization of human mesenchymal stem cells

Human MSCs were isolated from bone marrow of healthy donors in accordance with apceth’s GMP process using apceth’s proprietary medium. Cells were detached using TrypLE Select (Invitrogen). At the first passage, cells were collected and retrovirally transduced at MOI 1.5 using vectors that constitutively express HSV-TK or GFP transgene under control of the EFS promoter and a puromycin resistance gene. Subsequently transduced cells were enriched using 3 μg/ml puromycin (Sigma-Aldrich). After expansion, cells were cryopreserved at a density of 5x10^6^ MSCs/ ml.

The cryopreserved cells were analyzed with regard to surface marker expression, differentiation capacity and transgene expression. Adipogenic and osteogenic differention were analyzed with the StemMACS Media (Miltenyi Biotech) according to the manufacturers’ instructions. Transgene expression (GFP, HSV-TK) and the presence (CD73, CD90, CD105) and absence (CD19, CD34, CD45, HLA-DR) of surface marker were analyzed by flow cytometry (FC500, Beckman Coulter). After thawing the viability of MSCs was determined by Annexin V (eBioscience)/7AAD (Beckman Coulter) flow cytometry.

### Cell culture

The human glioblastoma cell lines U87 (American Type Culture Collection, Manassas, VA, USA) and NCE-G55T2 [6] and the murine glioblastoma cell line GL261 (DCTDC Tumor Repository) were cultured in MEMalpha (Invitrogen, Carlsbad, CA, USA) and Dulbecco’s modified Eagle’s medium (DMEM) (Invitrogen), respectively, supplemented with 2 mM L-glutamine, 2 mM sodium pyruvate, 100 U/ml penicillin, 100 μg/ml streptomycin, 0.25 μg/ml fungizone and 10% fetal bovine serum (Invitrogen). The multipotent neural stem cell line HB1.F3 has been extensively characterized in previous studies [16] and was maintained as adherent cultures in Dulbecco's modified Eagle medium (DMEM) supplemented with 2 mM L-glutamine and 10% fetal bovine serum (Invitrogen). The human L87 MSC cell line [35] was cultured in RPMI (Lonza) supplemented with 10% FBS (Biochrom) and 1% Glutamax (Gibco). All cells were maintained in tissue culture flasks in 5% CO_2_/95% air at 37°C in a humidified incubator and were routinely passaged at confluency. For the *in vivo* experiments, the cells were dispersed with a 0.05% solution of trypsin/EDTA (Invitrogen) or directly thawed, washed with phosphate-buffered saline (PBS) and adjusted to the final concentration in PBS. To obtain conditioned media, the cell lines U87, G55T2 and GL261 were grown on 35-mm plates to 80% confluency. Cultures were washed three times with serum-free medium and incubated for another 48 hours in serum-free DMEM low glucose supplemented with 2 mM L-glutamine. Conditioned media were collected, cleared by centrifugation for 10 minutes at 600g, and stored at -80°C. Cell labeling using the lipophilic tracer DiI (Molecular Probes, Eugene, OR, USA) was performed for 30 minutes according to the manufacturer’s protocol. *In vivo* tracking of the human mesenchymal stem cells L87 by MR imaging was enabled by labeling the cells with superparamagnetic iron oxide particles (SPIO) as described previously [7]. Briefly, SPIO (25 mg of Fe per milliliter; Micromod, Rostock, Germany) were added to poly-L-lysine (750 ng/ml) (Sigma, Munich, Germany) and mixed with medium at room temperature for 60 minutes. The medium of cultured MSC was then replaced with the freshly prepared labeling solution and incubated for 24 hours. Cellular internalization of SPIO particles was confirmed prior to intracerebral injection by Prussian blue staining.

### Gap junction assay

HSV-TK expressing MSCs (MSC_HSV-TK) were incubated with or without 100 μM gap junction inhibitor Carbenoxolone (Sigma) for 16-20h before cell detachment. Cells were harvested, washed in PBS and stained with 5 μM CMTPX (gap junction impermeable; Molecular Probes) and 1 μM Calcein AM (gap junction permeable; Molecular Probes) for 20 min at 37°C. After washing, MSCs were mixed with tumor cells at a ratio of 1:1 and were plated at a density of 1x10^5^ cells/24-well. To assess cell contact independent dye transfer, 5x10^4^ double stained MSC were seeded in the top well of a 96 transwell-plate, 5x10^4^ non-labeled tumor cells were placed in the bottom well. Cells were analyzed by flow cytometry directly after mixing and after 4h of (co-) cultivation to evaluate dye transfer of Calcein AM to unstained tumor cells.

### *In vitro* bystander assay

HSV-TK expressing MSCs (MSC_HSV-TK) and CMFDA-stained tumor cells (U87, G55T2, GL261) or GFP-expressing U87 cells (for ratio assay) were cocultured at a ratio of 1:1 or the indicated ratios. In all samples the tumor cell number was kept constant at 3.1x10^4^ per 12 well. Medium was changed to medium containing 100 μM ganciclovir (GCV) on day 1, 2 and 3 after seeding. Cocultures without GCV addition and tumor cells only were used as controls. The percentage of surviving tumor cells was analyzed by flow cytometry on study day 4, after addition of counting beads (Molecular Probes).

### *In vitro* migration

MSC migration in response to tumor-conditioned media was assessed using a modified Boyden chamber assay as previously described [15]. Briefly, quadruplicates of the chemoattractants were added to the lower wells of a 96-well modified Boyden chamber (Neuroprobe, Cabin John, MD), and wells were covered with an 8-μm pore size Nucleopore filter coated with 100 μg/ml collagen (Sigma). MSCs or HB1.F3 were then suspended at 2.5 or 1.5 x 10^4^ cells in 50 μl of serum-free DMEM medium containing 0.1% bovine serum albumin and seeded into the upper wells. After incubation for 5.5 hr at 37°C, nonmigrated cells were scraped off the upper side of the filter and filters were stained with Diff Quick (Dade, Switzerland). Nuclei of migrated cells were counted in ten high-power fields using a 20x objective with a calibrated ocular grid. Values were assessed in quadruplicate and expressed as the mean +/- standard deviation. The control migration was assessed in response to serum-free DMEM containing 0.1% bovine serum albumin only and reflects the basal migration rate of MSCs in this assay.

### *In vivo* studies

Orthotopic glioblastoma xenografts were established in 4- to 6-week-old male NMRI-nu/nu mice (Charles River, Sulzfeld, Germany). Mice were anesthetized (100 mg/kg ketamine and 5 mg/kg xylazine) and received a stereotactically guided injection of 2×10^5^ human U87 glioblastoma cells into the right forebrain (2 mm lateral and 1 mm anterior to bregma, at a 2.5 mm depth from the skull surface).

Ten days after tumor cell injection mice received a stereotactically guided injection of freshly dissociated 5×10^4^ or 2×10^5^ DiI-labeled, SPIO-loaded or eGFP-expressing MSCs into the left forebrain contralaterally to the growing tumor in the right forebrain and sacrificed 12, 13 or 17 days after tumor cell injection for assessment of MSC migration and tumor homing. Mice without glioma xenografts served as controls.

For therapeutic assessment freshly thawed 0.4×10^6^ HSV-TK expressing MSC were stereotactically injected to the tumor region two days after tumor cell application. Systemic prodrug treatment was performed by intraperitoneal injection of 50mg/kg GCV in 100μl for five days starting four days after initial tumor cell application. Tumor volume was assessed at day 20 by MR imaging. Survival was documented and animals were sacrificed at the onset of neurological deficits or significant weight loss in accordance with federal and institutional guidelines.

All animals were sacrificed by CO_2_ inhalation. The brain was removed, embedded in OCT, and stored at -80°C until further processed for histological analysis. All animal experiments were performed in accordance with federal and institutional guidelines and approved by the Institutional Animal Care and Use Committee.

### Magnetic resonance imaging (MRI)

Tumor growth and cell migration of SPIO-labeled MSC in intracerebral U87 human glioblastoma-bearing mice was evaluated on a 7T MR imaging system (ClinScan, Bruker, Ettlingen, Germany) [7]. Mice were anesthetized with 1% isoflurane (Baxter, Munich, Germany) in oxygen (0.5 l/min). Respiratory rates were monitored using a small animal vital sign monitor (SA Instruments Inc., Stony Brook, NY, USA). Coronal 2D T2 weighted turbo spin echo images were acquired to assess tumor location and size. Sequence parameter were: TE = 39 ms, TR = 2500 ms, BW = 250 Hz/pixel, turbo factor 7, matrix = 256x192, FOV = 20x15 mm^2^, 19 slices, 0.4 mm slice thickness with 0.1 mm gap. 3D susceptibility weighted imaging (SWI) was acquired in the same orientation to detect migration of SPIO-labeled cells. Sequence parameters were: TE = 7 ms, TR = 50 ms, FA = 15°, BW = 250 Hz/pixel, matrix = 192x144x72, FOV = 19x14x7.2 mm^3^. Images were analyzed on a viewing task card (Syngo MR B15, Siemens, Erlangen, Germany) running on the control unit of the MR system. Tumor volumes were quantified using GraphPad Prism Software (Version 4.03).

### Histological analysis

Frozen brains embedded in OCT were cut into serial 10 μm sections and fixed with 4% paraformaldehyde and mounted using Citifluor AF1 Glycerol/PBS (Science Services, Munich, Germany). The whole brain of tumor-bearing and control animals without brain tumors was evaluated for cellular migration and accumulation by analyzing DiI- or eGFP-positive cells in DAPI counterstained sections. Hot spots of intra- (IT) and peritumoral (PT) localized MSCs were quantified by counting the number of DiI- or eGFP-positive cells in ten randomly chosen high-power fields (1376 μm^2^ each) using a fluorescence microscope (Axioskop, Zeiss, Göttingen, Germany) and imaging Software (Axiovision AC 4.1, Zeiss). The peritumoral area (PT) was defined as a zone of 100 μm distance around the tumor border. SPIO-labeled cells were detected by Prussian-Blue staining. Briefly, cryosectioned brains were incubated with freshly prepared Perls’ reagent (1% potassium ferrocyanide, 3% hydrochloric acid, in distilled water) for 60 minutes and counterstained with neutral red.

### Statistical analysis

Differences between experimental groups were calculated by student's t-test or Mann–Whitney U Test and one way or ANOVA on Ranks using sigma-plot (Systat-Software Inc., Germany). All values were calculated as means ± standard deviation (SD) or median ± standard error (SE) as indicated with a p-value <0.05 considered statistically significant. Kaplan–Meier survival curves were statistically analyzed using log rank significance test (Aabel NG, Gigawiz, USA).

## Abbreviations

CMFDA: 5-Chlormethylfluoresceindiacetat
DAPI: 4',6-Diamidin-phenylindol
DiI: 1,1ʼ-Dioctadecyl-3,3,3ʼ,3ʼ-tetramethylindocarbocyaninperchlorat
EFS: embryonal fyn-associated substrate
FACS: fluorescence-activated cell sorting
GCV: ganciclovir
GFP: green fluorescent protein
GMP: good manufacturing practice
HA-tag: hemagglutinin-tag
HSV-TK: herpes simplex virus-thymidine kinase
IT: intratumoral
MR: magnetic resonance
MSC: mesenchymal stem cell
OCT: optimal cutting temperature
PT: peritumoral
SD: standard deviation
SE: standard error
SPIO: superparamagnetic iron oxide
SWI: susceptibility-weighted imaging
TRAIL: tumor necrosis factor related apoptosis inducing ligand
VEGF: vascular endothelial growth factor.
